# Conference Report: The Nour Foundation Georgetown University & Blackfriars Hall, Oxford University Symposium Series *Technology, Neuroscience & the Nature of Being: Considerations of Meaning, Morality and Transcendence Part I: The Paradox of Neurotechnology *8 May 2009

**DOI:** 10.1186/1747-5341-4-9

**Published:** 2009-07-17

**Authors:** Guillermo Palchik

**Affiliations:** 1 Interdisciplinary Program in Neuroscience, Georgetown University Medical Center Washington, DC, USA

## Abstract

This reviews the first of a tripartite symposia series dealing with novel neuroscientific technologies, the nature of consciousness and being, and the questions that arise from such interactions. The event took place on May 8 2009, at Georgetown University, and brought together ten leading figures on fields ranging from Neuroscience and Robotics to Philosophy, that commented on their research and provided ethical, moral and practical insight and perspectives into how these technologies can shape the future of neuroscientific and human development, as well as denoting the potential abuses and the best way to proceed about them.

## Introduction

On May 8, 2009, The Nour Foundation, a public charitable organization and NGO in special consultative status to the United Nations, together with Georgetown University, Blackfriars Hall at Oxford University, and the Potomac Institute for Policy Studies' Center for Neurotechnology Studies, presented *The Paradox of Neurotechnology*, a thought-provoking symposium exploring the technologies of neuroscience and the ways in which these tools and methods can lead to a deeper understanding of the nature of being. The discussions brought together leaders in the field to address the pressing issues, questions, and potential dilemmas arising in and from the use of neurotechnologies, and how such progress prompts deeper neuroethical considerations. The symposium was the first in a three-part series, with subsequent meetings to be held at the University of Oxford on 22 July 2009, and the United Nations in New York on 11 September 2009. Further information regarding the symposium can be found on their website [1].

The symposium began with an introduction and keynote by Prof. James Giordano, Professor of Neuroscience, Ethics, and Philosophy at the Institute for Psychological Sciences, Centre for Philosophical Psychology; Fellow, Blackfriars Hall, University of Oxford; Director of the Center for Neurotechnology Studies and Chair of Academic Programs at the Potomac Institute for Policy Studies in Virginia; and former Samueli-Rockefeller Professor of Medicine and Neuroscience and Director of the Program for Brain, Mind and Healing Research at the Georgetown University Medical Center, where he maintains an adjunct professorship. Giordano set the tone by positing that as we approach the frontier realms of science, we encounter new and novel possibilities that require us to deal with the contingencies arising not only from what is yet unknown, but what may ultimately remain unknowable. He outlined the basic premises of a philosophy of science to address and unify domains of metaphysics, epistemology, anthropology and ethics, and proposed a paradigm for precautionary progress that involves a pragmatically balanced optimism grounded in proactive responsibility.

Following Prof. Giordano, Dr. Erik Parens, Senior Research Scholar at the Hastings Center and Visiting Professor at Sarah Lawrence College, focused on the ways in which technologies such as fMRI form our self-images to address the benefits and dangers scientists encounter when relying on the use of novel and emerging techniques. Parens spoke to the issues surrounding definitions of normality, and how these constructs can affect the way in which we treat those who fall outside of the artificial, and sometimes arbitrary, distinctions and boundaries drawn while describing what is acceptable or desirable.

Dr. Dennis McBride, President Emeritus of the Potomac Institute for Policy Studies and adjunct professor at Georgetown University, raised the question of whether contemporary psychology as a discipline is capable of accommodating the conditions required for reconciling brain science with studies of the mind. McBride's thesis calls for an integrative *neuroethology *that allows for both objective assessment and the possibility for correlation of more subjective aspects of cognition (such as emotion, motivation, intentionality) to biological and environmental variables.

Dr. Layne Kalbfleisch, Assistant Professor in Educational Psychology at the College of Education and Human Development at George Mason University and Pomata Term Professor of Cognitive Neuroscience at George Mason's Krasnow Institute for Advanced Study, provided an overview of the ways in which neurotechnologies such as fMRI and other forms of neuroimaging have become increasingly representative of "real world" cognition. Kalbfleisch elucidated what she has called "neuro-myths" that reflect misconceptions held on the part of both the public and some members of the academic community about the capacities and limitations of neurotechnologies, and, by extension, the brain-mind. Illustrating the recent surge in mass market books addressing the brain, mind and cognitive capacity, she advocated a more explicit and direct communication of how abilities and constraints of neurotechnology allow and/or restrict insight to, and predictions about, the potential of the brain-mind, and those fields that employ this knowledge in research and everyday applications.

In a compelling lecture, Dr. Kevin FitzGerald, Associate Professor of Molecular Genetics and David Lauler Chair of Catholic Healthcare Ethics at Georgetown University, dealt with the notion of "personalized medicine" as applied to neurogenetics, and raised questions of how to balance the progressive ubiquity of individual patients' information in an increasingly accessible medical data system with libertarian concerns and the need for privacy. As well, FitzGerald addressed the question of "what we really know and don't know" about the capacities and limits to categorize and/or change the self, as based upon neurogenetic data. In this light, he discussed the process of patients' informed consent and the ethical dilemmas that could occur as we respond to escalating socio-economic pressures to "use what we've got" in practical applications. Here again, the treatment-enhancement/normality debate was brought to the fore, and the core theme of linking epistemology to an applied ethic of research and clinical care was highlighted.

Dr. Jeffrey Krichmar, Assistant Professor in the Department of Cognitive Sciences at the University of California, Irvine, shared his cutting-edge work on robotics and neural networks. Krichmar highlighted work on artificially created machines that approach decisional-processes which may ultimately obtain the parameters of human capacity, emphasizing that we should consider these potentialities beyond the mere utility of robots, for it may be that as a consequence of such advanced and iterative synthetic neural network development robots (and perhaps other computational devices) will be able to manifest some form of consciousness. At first impression, this would allow a deeper insight to the mechanisms of consciousness, albeit not necessarily the same substrates as those of human or animal consciousness per se, but upon further reflection also instantiates moral consideration of what these "conscious" devices may feel, and how such sentience might dictate how we treat machines.

Dr. Susan Schneider, Assistant Professor in the Department of Philosophy at the University of Pennsylvania and an affiliated faculty member at the Center for Cognitive Neuroscience and the Institute for Research in Cognitive Science, approached the topic of neurotechnology and its implications and effects on humanity from a considerably deeper metaphysical standpoint. Working to afford insights to the trans-human potential of any neurotechnology, Schneider addressed how ontological values may need to be readdressed and perhaps reconsidered as we move toward increasingly integrative use and employment of neurotechnological devices on a variety of levels, from the most sublime to the extreme. Moreover, Schneider's thesis – that pervasiveness and progression of the technologic trend mandates not only philosophical reflection, but evaluation of the ways this will define the human person and human condition in practical terms – in many ways reflects and frames issues of neurotechnology in both the larger treatment-enhancement debate and the entire trans-humanist movement, both of which constitute contentious, provocative, and important aspects of the discourse.

Also presenting from the University of Pennsylvania was Dr. Sheri Alpert, Lecturer and Associate Fellow at the Center for Bioethics, who discussed the ethical conundrums that arise with novel medical technologies, specifically those that affect the brain-mind and the subjective dimensions of emotion, consciousness and intention. Of particular focus was the development and use of new neuropsychopharmacological agents in light of extant ambiguities related to: 1) the actual definition of normality as relates to brain-mind function and its manifestations in various social contexts; 2) the mechanisms of action and the effect of these agents, as based upon a contingent understanding of neural function, and 3) the treatment-enhancement question, writ both small (viz., in individuals) and large (viz., as a social construct and practical "kind," reflective of a somewhat Szaszian perspective). To illustrate, Dr. Alpert constructed a hypothetical case scenario that addressed the actions and effects of a fictional psychopharmacologic agent, and how social values systems are exploited to justify use and misuse. Her lecture highlighted the ways in which social and market forces can influence scientific funding, development and application(s), and how this is an important factor to sustain the obligation to consider and solicit bioethical insight and analyses at all stages of neurotechnology research and development.

In a most thought-provoking, if not provocative, lecture Dr. Sam Parnia, Clinical Research Fellow at the Weill Cornell Medical School in New York and Founder and Director of the Human Consciousness Project at the University of Southampton in the UK – a multidisciplinary collaboration of international scientists and physicians who have joined forces to research the nature of consciousness and its relationship with the brain – described his studies examining conscious processes during cardiac arrest. Without doubt, these studies present numerous challenges and potentially open new vistas from a neuroscientific perspective. Although affording insight to the mechanistic events and phenomenal experiences of consciousness during the dying process, such insights raise other, more ontological (if not cosmological) questions about the nature of consciousness, self, and the process of life and death as they relate to a philosophy and perhaps physics of the natural universe, prompting a variety of ethical questions and potential dilemmas.

Finally, renowned philosopher Prof. Roger Scruton of the Institute for the Psychological Sciences in Virginia and the Centre for Philosophical Psychology at Blackfriars Hall, Oxford University, presented a compelling lecture in which he addressed and considered the ways in which our cultural and political beliefs affect the scope and conduct of neuroscientific research and its applications in society. Scruton argued that while the neuroscientific approach is necessary, it is not, nor should it be, sufficient for a "complete" assessment and regard for the human person, and/or other organisms. Scruton warned against the overuse and misuse of neuroscientific information, cautioned against mereological thinking, and urged awareness of the misconception that any and all new data constitute "knowledge." In suggesting that we prescind, Scruton called for an acknowledgment of the "limits of both technology and knowledge," and invoked a collective intellectual honesty that accepts what is known and what is yet to be discovered.

Overall, the symposium presented the issue of neurotechnology holistically in a form that allowed for open thought and dialogue and a broadening view of science, scientists, and technology – not in isolation, but as constituents of social culture(s) – and the direction in which neurotechnological progress might and perhaps should progress in the future. Rather than being over-indulged with a single perspective as *the *truth, the symposium reinforced the tangential nature of much of what we know about brains, minds, and consciousness, though given the inertia of neurotechnology we now have an ever-widening opportunity to explore many, and perhaps partial, truths. As summarized by Dr. Giordano in his concluding remarks: "We must recognize that, as foretold by Lenk and Jonas, the status quo *is *progress. This pendulum of progress cannot be impeded, nor will it be reversed. And perhaps it should not be. The goal, therefore, is to recognize our responsibility to engage the profound ethical, social and legal implications of the truths we may seek and reveal about the brain and mind through our use of technology, and acknowledge its potential uses as well as misuses in both neuroscience and society. To do so will require that we identify the gaps in knowledge and safety, analyze how such gaps can incur burdens, threats and risks, and work to ensure that precaution is taken so as not to be exclusory of technology, but to advance with humanitarian concern and prudence," which speaks to the focus of the symposium series on the whole.

The next meeting, "*Brain, Mind & the Nature of Being*," to be held at the Ioannou Centre for Classical Studies, Oxford, England on 22 July specifically addresses those ways in which neuroscience and neurotechnology have, have not, and perhaps cannot reveal aspects of the human condition such as aesthetics, social dynamics, and spirituality, and how the construct of transcendence may be intrinsic to any consideration of the brain-mind, at least as we currently know it. This symposium will be followed by a conference on September 11 at the United Nations, "*Toward a Common Morality*" that will address the neural basis of moral sense and decision making, and how such processes may illustrate a common basis for moral consideration and human "good."

Ultimately, as Dr. Joseph Salim, Vice-President of the Nour Foundation, aptly stated: "As technological advances in neuroscience continue to demonstrate that human beings share a common physical and neurological constitution, it increasingly behooves us to explore the scientific underpinnings of seemingly universal emotional and psychological behaviors if we are to work toward developing a more holistic and integrated understanding of the human condition."

## Abbreviations

fMRI: functional magnetic resonance imaging.

## Competing interests

The author declares that they have no competing interests.
